# The oxytocin receptor rs2254298 polymorphism and alcohol withdrawal symptoms: a gene–environment interaction in mood disorders

**DOI:** 10.3389/fpsyt.2023.1085429

**Published:** 2023-07-14

**Authors:** Guanghui Shen, Shizhuo Yang, Liujun Wu, Yingjie Chen, Yueling Hu, Fan Zhou, Wei Wang, Peining Liu, Fenzan Wu, Yanlong Liu, Fan Wang, Li Chen

**Affiliations:** ^1^Wenzhou Seventh People’s Hospital, Wenzhou, China; ^2^School of Mental Health, Wenzhou Medical University, Wenzhou, China; ^3^School of Pharmacy, Wenzhou Medical University, Wenzhou, China; ^4^Applied Psychology (Ningbo) Research Center, Wenzhou Medical University, Ningbo, China; ^5^Cixi Biomedical Research Institute, Wenzhou Medical University, Ningbo, China; ^6^Department of Pediatrics, The Second Affiliated Hospital and Yuying Children's Hospital of Wenzhou Medical University, Wenzhou, China; ^7^Laboratory of Translational Medicine, Affiliated Cixi Hospital, Wenzhou Medical University, Ningbo, China; ^8^Beijing Hui-Long-Guan Hospital, Peking University, Beijing, China; ^9^Key Laboratory of Psychosomatic Medicine, Inner Mongolia Medical University, Hohhot, Inner Mongolia, China; ^10^Zhejiang Provincial Clinical Research Center for Mental Disorders, The Affiliated Wenzhou Kangning Hospital, Wenzhou Medical University, Wenzhou, China

**Keywords:** alcohol use disorder, alcohol withdrawal, mood disorders, single-nucleotide polymorphism, OXTR

## Abstract

**Objective:**

Alcohol use disorder (AUD) is a common mental disorder characterized by repeated withdrawal episodes. Negative emotions during withdrawal are the primary factors affecting successful abstinence. Oxytocin is a critical modulator of emotions. OXTR, the oxytocin receptor, may also be a promising candidate for treating alcohol withdrawal symptoms. Previous studies indicated that people with different genotypes of OXTR rs2254298 were reported to suffer from more significant depressive or heightened anxiety symptoms when experiencing early adversity. The present study aims to explore the modulatory role of the polymorphism OXTR rs2254298 on mood disorders during alcohol withdrawal and to help researchers better understand and develop effective relapse prevention and interventions for alcohol use disorders.

**Methods:**

We recruited 265 adult Chinese Han men with AUD. Anxiety and depressive symptoms were measured using the Self-Rating Anxiety Scale and Self-Rating Depression Scale. Alcohol dependence levels were measured using Michigan Alcoholism Screening Test. Genomic DNA extraction and genotyping from participants’ peripheral blood samples.

**Result:**

First, a multiple linear regression was used to set the alcohol dependence level, OXTR.rs2254298, interaction terms as the primary predictor variable, and depression or anxiety as an outcome; age and educational years were covariates. There was a significant interaction between OXTR rs2254298 and alcohol dependence level on anxiety (*B* = 0.23, 95% confidence interval [CI]: 0.01–0.45) but not on depression (*B* = −0.06, 95% CI: −0.30 – 0.18). The significance region test showed that alcohol-dependent men who are GG homozygous were more likely to experience anxiety symptoms than subjects with the A allele (A allele: *β* = 0.27, *p* < 0.001; GG homozygote: *β* = 0.50, *p* < 0.001). Finally, re-parameterized regression analysis demonstrated that this gene–environment interaction of OXTR rs2254298 and alcohol dependence on anxiety fits the weak differential susceptibility model (*R^2^* = 0.17, *F* (5,259) = 13.46, *p* < 0.001).

**Conclusion:**

This study reveals a gene–environment interactive effect between OXTR rs2254298 and alcohol withdrawal on anxiety but not depression. From the perspective of gene–environment interactions, this interaction fits the differential susceptibility model; OXTR rs2254298 GG homozygote carriers are susceptible to the environment and are likely to experience anxiety symptoms of alcohol withdrawal.

## 1. Introduction

China’s alcohol market has grown rapidly over the past 30 years to become one of the world’s largest, accompanied by significantly increased consumption (1). According to the World Health Organization, alcohol consumption leads to 3 million deaths each year globally and causes disabilities and poor health in millions of individuals. Excessive alcohol use accounts for 5.1% of the global disease burden (2). Alcohol use disorder (AUD) is the second most common mental disorder, following depression (3, 4). It is a maladaptive pattern of alcohol use characterized by repeated and heavy drinking, cognitive impairment, and a range of symptoms of alcohol withdrawal (5, 6). Alcohol withdrawal has a set of characteristic symptoms that occur when an alcohol-dependent person suddenly stops or reduces the use of alcohol; this behavior can trigger a stress response in the brain and lead to a sudden increase in anxiety and depression (7).

Withdrawal from chronic alcohol exposure is a potent stressor that can affect the functional integrity of the HPA axis and is strongly associated with mood disorders, which may lead to withdrawal failure and relapse into AUD (8–12). However, the literature on alcohol withdrawal suggests that not all individuals experience depression or anxiety in the context of alcohol withdrawal, which suggests a potential physiological mechanism that contributes to alleviate negative emotions during withdrawal and improve the success rate of alcohol abstinence (13). Genetic studies linked individual differences to specific allelic variants arising from single-nucleotide polymorphisms (14). However, single genetic polymorphisms do not act independently, but rather can interact with environmental factors on cognition, emotion, and behavior (14). Gene–environment (G × E) interactions are defined as different gene carriers’ differential risk/susceptibility to environmental exposures. Current theoretical gene–environment interaction models include the diathesis-stress and differential susceptibility models. The diathesis-stress model assumes that individuals with risk genes are more likely to develop psychosocial problems in poor situations (15). Based on evolutionary theory, differential susceptibility models emphasize genetic susceptibility, with susceptibility gene carriers being more environmentally sensitive (15).

The oxytocin (OXT) system is a potential target for AUD treatment (16). OXT is a nonapeptide hormone initially known for its role in breastfeeding and childbirth (17). In recent years, the research focus on OXT shifted to regulating human social emotions and related behaviors (18). OXT can regulate several negative emotions and attention mechanisms, which is mediated by the oxytocin receptor (OXTR) expressed on the target neuronal cells (19). The OXTR is a peptide consisting of 389 amino acids located on the short arm of chromosome 3 with three introns and four exons (20). The OXTR gene was associated with social cognition and behavior in specific populations (21–23), particularly concerning its relationship to vulnerability and psychiatric disorder treatment (24). The literature suggests that polymorphic variation of the OXTR impacts human behavior and social cognition (25). During human evolution, comparative genetics suggest that mutations replace guanine (G) with adenine (A) (26). Rs2254298 is situated in the third intron of the OXTR gene, and this single-nucleotide polymorphism (SNP) of OXTR is associated with emotion (27). Girls with the rs2254298 AG genotype reported more significant depressive and heightened social and physical anxiety symptoms when experiencing early adversity (24). Incarcerated Chinese men with the GG genotype of OXTR rs2254298 had a higher vulnerability to the effect of childhood adversity on depressive symptoms. Recent studies showed that the genetic variant of OXTR rs2254298 influences the neurobiology of attention-deficit hyperactivity disorder and anxiety, leading to more significant functional, social, and emotional impairment (28). While these studies support OXTR genotypes conferring greater vulnerability for psychopathology in adverse environments, it is unclear whether genetic variants in OXTR moderate alcohol withdrawal-related anxiety and depression.

OXTR could be a candidate gene for modulating negative emotions during withdrawal. Genetic polymorphisms of OXTR could contribute modestly to individual differences during alcohol withdrawal. However, the interaction between alcohol withdrawal and the OXTR gene polymorphism on anxiety and depression remains unclear. Therefore, this study focused on the interactive effects of OXTR rs2254298 and alcohol withdrawal on depressive or anxiety symptoms, exploring protective genotypes/susceptibility genotypes.

## 2. Materials and methods

### 2.1. Participants

We recruited 265 Chinese Han men from several hospitals in northern China. The inclusion criteria for alcohol-dependent participants were as follows: (1) diagnosis of alcohol dependence by at least two trained psychiatrists based on DSM-IV; (2) sufficient literacy skills; (3) Han ethnicity. The exclusion criteria were as follows: (1) history of other substance abuse or dependence (excluding nicotine); (2) cardiovascular, liver, or kidney disease; (3) participants or their first-degree relatives with a history of severe psychiatric disorders. The mental health status of all patients undergoing alcohol withdrawal have been evaluated by the experienced psychological counselor prior hospitalization and the subsequent mandatory 3-weeks detoxification. Thus, patients that have pre-existing major depression or anxiety disorders were excluded in the current study based on the evaluation conducted by the experienced psychological counselor. The Institutional Review Board of the Inner Mongolian Medical University approved the study. The participants undergone mandatory detoxification for at least 3 weeks and then were asked to complete a series of questionnaires and provide a blood sample, which was stored at −80°C for DNA extraction. All patients provided written informed consent and were told the blood sample would be subjected to a gene assay.

We considered utilizing continuous variables to quantify the educational levels, which were then applied as covariates in a linear regression analysis to gain a deeper insight into potential influences. We also supplemented the boundaries of high/middle/low education in the Chinese system with an academic years classification: ≤ 9 years as low education; 9 years < middle education ≤12 years; 12 years < high education. The average educational years of the participants in the study was 10.30 ± 2.82 years, with 111 (41.89%) having low educational levels (≤9 years), 105 (39.62%) having middle educational levels (>9 and ≤ 12 years), and 49 (18.49%) having high education levels (> 12 years).

### 2.2. Measures

#### 2.2.1. Genotyping

Genomic DNA was extracted from 5 mL of peripheral blood from each participant using standard techniques. The OXTR rs2254298 SNP was genotyped using 5′ nuclease fluorescent TaqManTM primers (Applied Biosystems, Foster City, CA). Reactions were carried out according to the manufacturer’s protocol. All laboratory procedures were carried out in a manner blind to case–control status. The conditions of PCR were as follows: 50°C for 2 min, 95°C for 10 min, followed by 50 cycles of 95°C for 15 s and 60°C for 1 min. Ten percent of the DNA samples were duplicated randomly and tested, and no-fault genotyping was found.

#### 2.2.2. Alcohol dependence level

Alcohol dependence level was measured using the Michigan Alcoholism Screening Test (MAST) (29), a questionnaire containing a 25-item self-report in which respondents rate the severity of dependence-related alcohol use behaviors (30). The test uses a four-point scale from 1 (not at all) to 4 (very much). The scale has high internal consistency with a Cronbach’s α of 0.90 (30).

#### 2.2.3. Anxiety

Anxiety was measured by the Zung Anxiety Self-Assessment Scale (SAS), a 20-item scale that covers a wide range of anxiety symptoms, from mental to physical. The questionnaire uses a four-point Likert scale ranging from 1 (none or a small amount of the time) to 4 (most or all the time). Higher total scores indicate more severe anxiety symptoms. The SAS has satisfactory psychometric properties with a Cronbach’s α of 0.82 (31).

#### 2.2.4. Depression

Depression was measured using Zung Self-Rating Depression Scale (SDS), which contains 20 items. Each item is rated on a four-point Likert scale (from 1 = “rarely or none of the time” to 4 = “most or all of the time”). Higher total scores indicate more severe symptoms of depression. The SDS has internal consistency with a Cronbach’s α of 0.79 (32).

### 2.3. Data analysis

All analyzes were performed using R software (R version 4.0.2). The *χ*^2^ test was used to determine whether the genotype distribution of OXTR rs2254298 followed Hardy–Weinberg equilibrium and assessed the association between OXTR rs2254298 polymorphisms and susceptibility to AUD. Pearson and Spearman’s correlations were conducted to determine the associations among OXTR rs2254298, age, academic years, alcohol dependence level, anxiety, and depression.

Multiple linear regression was used as a preliminary exploration to test for significant gene–environment interactions (G × E). When significant interactions were found, we used region of significance (RoS) analysis to examine the forms of interaction effects. Based on the simple slope analyzes, this approach generates potential thresholds where the association between gene and alcohol dependence level is significant for estimating the forms of G × E interaction.

Finally, a re-parameterized regression model was used to test the pattern of G × E interaction as follows (15):


$$Y=\left\{\begin{array}{c} Group:D=0B_{0}+B_{1}\left(X-C\right)B_{3}X_{2}+B_{4}X_{3}+E\\ Group:D=1B_{0}+B_{2}\left(X-C\right)B_{3}X_{2}+B_{4}X_{3}+E \end{array}\right\}$$

Where Y is the outcome variable of anxiety and depression (standard normalization), group is the OXTR polymorphism group, X is the MAST score (standard normalization), X2 and X3 are covariates (age and academic years), and C is the crossover point where the slopes of different genotypic subgroups cross. C and its 95% confidence interval were the initial criteria for judging the mode of interaction. If the point estimate and 95% confidence interval estimate fall at the maximum MAST score, the interaction fits the diathesis-stress model. Conversely, the forms of interaction fit the differential susceptibility model. To clarify the patterns of the interaction, the models were subdivided into a strong/weak differential susceptibility model and a strong/weak diathesis-stress model. Strong models assume that only individuals carrying the risk/plasticity allele are sensitive to the environment, and those carrying the non-risk/non-plasticity allele are unaffected by the environment. The weak version assumes that carriers of both alleles are sensitive to the environment; however, carriers of the risk/plasticity allele are more sensitive than those carrying the non-risk/non-plasticity allele. The F-test (for nested models), the Akaike information criterion, and the Bayesian information criterion (for non-nested models) were used to determine which model fits best.

## 3. Results

### 3.1. Descriptive statistics

Descriptive statistics of research variables are displayed in Table 1. This study included 265 participants with a mean age of 45.58 ± 9.20 years. Participants’ mean education years was 10.30 ± 2.82 years; there were 111 (41.89%) low education (≤ 9 years), 105 (39.62%) middle education (>9 and ≤ 12 years), and 49 (18.49%) high education(>12 years).

**Table 1:** Descriptive statistics (*n* = 265).

Variables	Mean ± SD
Age	45.58 **±** 9.20
Education years	10.30 **±** 2.82
Anxiety symptoms	35.44 **±** 8.86
Depression symptoms	56.98 **±** 10.32
MAST score	10.71 **±** 5.00

Alcohol dependence level is measured by Michigan Alcoholism Screening Test (MAST).

The genotype frequencies for OXTR rs2254298 were AA: 8.7%, AG: 43.4%, and GG: 47.9%, indicating that OXTR rs2254298 accorded with Hardy–Weinberg equilibrium (*χ^2^* = 0.18, *df* = 2, *p* > 0.05; Table 2). The minor allele frequency of this SNP was 30.3%, consistent with the HapMap and 1,000 genomes frequencies (0.30–0.34; Table 3). Participants were grouped as GG homozygote and A allele carriers according to their OXTR rs2254298 genotype (recode: A allele =0, GG homozygote = 1) (33, 34).

**Table 2:** Hardy–Weinberg equilibrium in participants.

Genotype	Number of people		Percentage
AA	23		8.7%
AG	115		43.4%
GG	127		47.9%
*χ^2^*	0.18	*p*	0.67

**Table 3:** Genotype frequency of OXTR rs2254298 in different populations.

	gnomAD			1000Genomes		
	AA	AG	GG	AA	AG	GG
European	1.1%	19.1%	79.7%	1.0%	19.7%	79.0%
American	4.3%	32.8%	62.7%	4.9%	37.4%	60.4%
East Asian	9.4%	42.5%	48.2%	11.6%	44.9%	43.4%

Table 4 displays correlations among research variables. OXTR rs2254298 was not significantly correlated with MAST, SAS, or SDS scores. The independent sample t-test showed no significant difference between genotypic groups in MAST, anxiety, or depression scores (MAST: *t* = −0.60, *p* = 0.55; Anxiety: *t* = 0.43, *p* = 0.067; Depression: *t* = −1.30, *p* = 0.19; Table 5). SAS and SDS scores were positively correlated with MAST scores (*r* = 0.38, *p* < 0.001; *r* = 0.21, *p* < 0.001).

**Table 4:** Descriptive statistics and correlations among study variables.

	rs2254298	Age	Education years	Alcohol dependence level	Anxiety symptoms	Depression symptoms
rs2254298	1					
Age	0.04	1				
Education Years	0.01	−0.30^***^	1			
Alcohol dependence level	0.01	−0.01	0.06	1		
Anxiety symptoms	−0.03	0.09	0.04	0.38^***^	1	
Depression symptoms	0.08	0.05	−0.01	0.21^***^	0.21^***^	1

^*^p < 0.05; ^**^p < 0.01; ^***^p < 0.001.

**Table 5:** Independent sample test.

rs2254298 polymorphism	Age	Education years	Alcohol dependence level	Anxiety symptoms	Depression symptoms
A allele	45.44 (8.77)	10.09 (2.88)	10.54 (5.08)	35.67 (8.11)	56.19 (10.84)
GG homozygote	45.73 (9.66)	10.53 (2.75)	10.91 (4.93)	35.20 (9.63)	57.84 (9.70)
*t*	−0.26	−1.25	−0.60	0.43	−1.30
*p*	0.80	0.21	0.55	0.67	0.19

### 3.2. The interactions of alcohol dependence level and OXTR rs2254298 for anxiety

Traditional hierarchical regression analysis was conducted to identify the interaction between OXTR rs2254298 and alcohol dependence level for anxiety symptoms. Alcohol dependence level significantly affected anxiety scores (*β* = 0.38, *p* < 0.001), such that a higher alcohol dependence level was associated with higher anxiety scores. There was no significant effect of OXTR rs2254298 on anxiety symptoms (*β* = 0.04, *p* = 0.54). In step 3, the interaction of alcohol dependence level and OXTR rs2254298 was included in the regression equation. The interaction of alcohol dependence level and OXTR rs2254298 accounted for a significant portion of the variance in anxiety symptoms (*β* = 0.16, *p* < 0.05; Table 6). For the interaction for anxiety, the partial correlation value of 0.13 and the semi-partial correlation value of 0.12 indicated that 1.4–1.7% of the variance in anxiety could be explained by alcohol dependence level × OXTR rs2254298 interaction.

**Table 6:** Interaction between rs2254298 and alcohol dependence level on anxiety.

	Variables	Anxiety symptoms					
		*ΔR^2^*	*B*(*SE*)	*β*	*t*	*p*	95%CI
Step1	Age	0.01	0.01 (0.01)	0.12	1.85	0.06	[0.01, 0.03]
	Education years		0.03 (0.02)	0.08	1.24	0.22	[0.02, 0.07]
Step2	Alcohol dependence level	0.15	0.38 (0.06)	0.38	6.65	< 0.001	[0.27, 0.49]
	rs2254298		0.07 (0.11)	0.04	0.61	0.54	[0.16, 0.29]
Step3	Alcohol dependence level× rs2254298	0.01	0.23 (0.11)	0.16	2.03	0.04	[0.01, 0.45]

**Figure 1:**
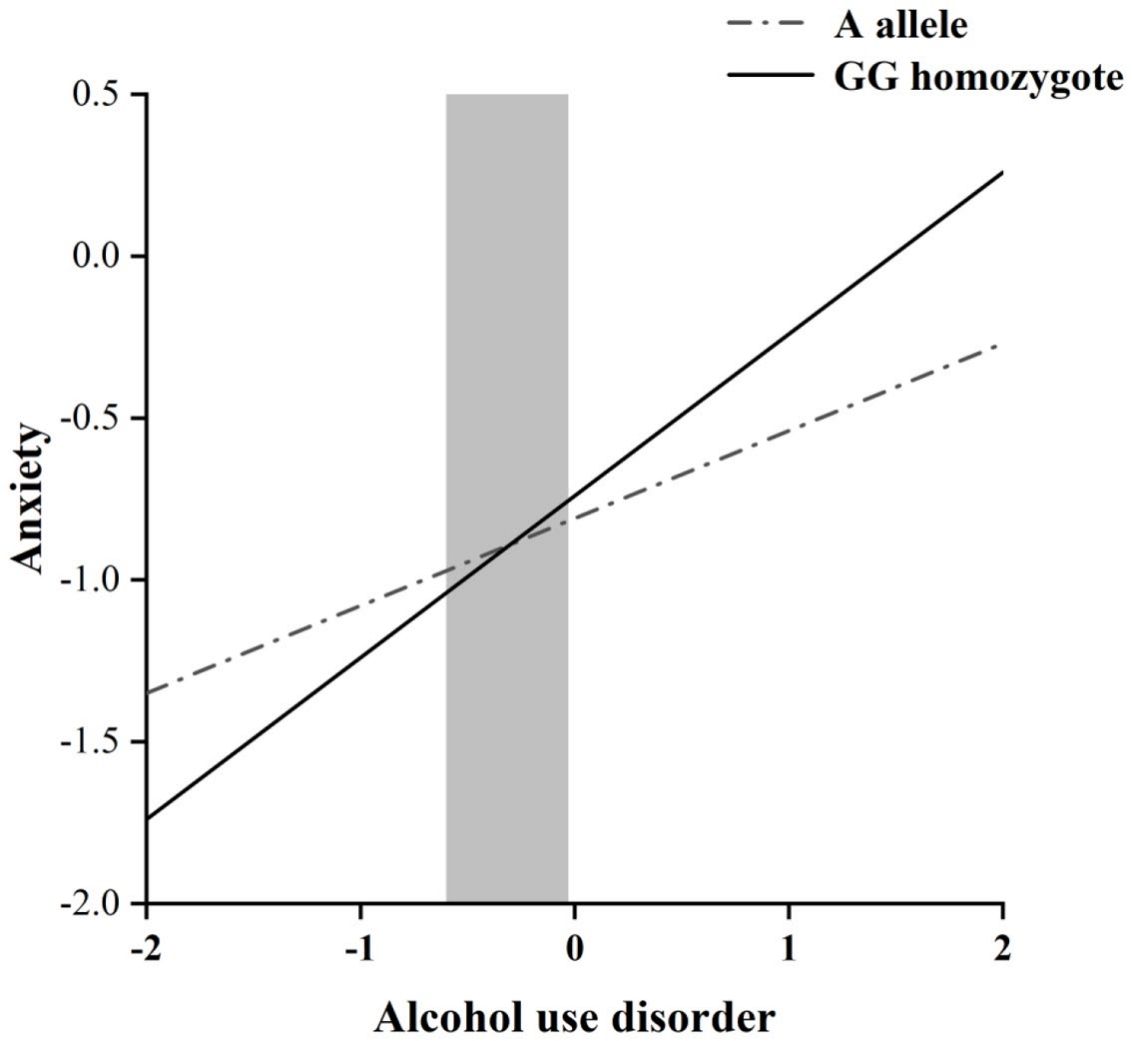
RoS test of the simple slopes on anxiety from alcohol dependence level in the OXTR rs2254298 allelic group. The gray shaded area represents the 95% confidence interval of the crossover point C of the interaction on the alcohol use disorder severity axis, 95% confidence interval of the crossover point C ranging from −0.60 to −0.03. Simple slope at A allele = 0.27, *t* = 8.60, *p* < 0.001. Simple slope at GG homozygote = 0.50, *t* = 10.72, *p* < 0.001.

The RoS test was performed to interpret the interaction effect. The simple slopes for alcohol dependence level on anxiety were: A allele carriers: *β* = 0.27, *t* = 8.60, *p* < 0.001; GG homozygote carriers, *β* = 0.50, *t* = 10.72, *p* < 0.001; Crossover point on predictor = − 0.30. The lower and upper bounds of regions of significance were − 0.60 and − 0.03, respectively, suggesting that GG homozygous subjects would be more likely to experience high anxiety symptoms than subjects with the A allele (Figure 1).

### 3.3. The interactions of alcohol dependence level and OXTR rs2254298 for depression

The same analysis was performed for depression symptoms (Table 7). Alcohol dependence level significantly affected depression scores (*β* = 0.21, *p* < 0.001), such that a higher alcohol dependence level indicates a higher level of depression symptoms. Similarly, OXTR rs2254298 was not significantly associated with depression symptoms directly (*β* = −0.07, *p* = 0.23). However, unlike the anxiety symptoms, alcohol dependence level and OXTR rs2254298 had non-significant interaction in depression symptoms (*β* = −0.04, *p* = 0.16). This exploratory analysis showed no significant interaction between alcohol dependence level and OXTR rs2254298, and thus depression symptoms were not considered in the subsequent analyzes.

**Table 7:** Interaction between rs2254298 and alcohol dependence level on depression.

	Variables	Depression symptoms					
		*ΔR^2^*	*B*(*SE*)	*β*	*t*	*p*	95%CI
Step1	Age	0.001	0.005 (0.01)	0.05	0.74	0.46	[−0.01, 0.02]
	Education Years		0.001 (0.02)	0.002	0.03	0.98	[−0.04, 0.05]
Step2	Alcohol dependence level	0.05	0.21 (0.06)	0.21	3.38	< 0.001	[0.08, 0.32]
	rs2254298		−0.14 (0.12)	−0.07	−1.20	0.23	[−0.38, 0.09]
Step3	Alcohol dependence level× rs2254298	0.01	−0.06 (0.12)	−0.04	−0.52	0.61	[−0.30, 0.18]

### 3.4. Re-parameterized regression analysis

We performed a re-parameterized regression analysis to test a specific pattern of alcohol dependence level **×** OXTR rs2254298. The weak differential susceptibility (Model B) explained a significant amount of variance in anxiety (*R*^2^ = 0.17, *p* < 0.001; Table 8). The crossover point C estimated = −0.30, where the slopes from alcohol dependence level to anxiety in the A allele group (*B*_1_ = 0.27, *SE* = 0.08, *p* < 0.001) and GG homozygote group (*B*_2_ = 0.50, *SE* = 0.08, *p* < 0.001) were significant. Based on Model B, constraining *B*_1_ = 0 led to Model A (strong differential susceptibility); fixed C to the maximum of MAST scores led to Model C (strong diathesis-stress) and Model D (weak diathesis-stress). This study used the F-test with Akaike information criterion and Bayesian information criterion values for model comparison. Compared with Models A and D, Model B added one more parameter and explained more variance (Model A: *ΔR*^2^ = 0.04, *F* = 10.39, *p* < 0.001, Model D: *ΔR*^2^ = 0.01, *F* = 3.67, *p* < 0.05). Compared with Model C, Model B added two more parameters and explained more variance (*ΔR*^2^ = 0.12, *F* = 32.93, *p* < 0.001). All statistical indexes support Model B (i.e., weak differential susceptibility), in which the A allele was a non-plasticity allele, and the GG homozygote was a plasticity homozygote.

**Table 8:** Results for re-parameterized regression model for anxiety.

Parameter	Differential susceptibility		Diathesis-stress	
	Strong model A	Weak model B	Strong model C	Weak model D
*B_0_*	−0.88 (0.43)	−0.89 (0.45)	0.95 (0.44)	1.60 (0.43)
*B_1_*	0.00 (−-)	0.27^***^(0.08)	0.00 (−-)	0.35 (0.06)^***^
*C*	−0.11 (0.23)	−0.30 (0.51)	1.94(−-)	1.94 (−-)
95%CI of C	[−0.56, 0.34]	[−1.30, 0.70]	(−-)	(−-)
*B_2_*	0.50 (0.08)^***^	0.50 (0.08)^***^	0.15 (0.04)^**^	0.41 (0.06)^***^
*B_3_*	0.02 (0.02)	0.02 (0.02)	0.02 (0.02)	0.02 (0.02)
*B_4_*	−0.01 (0.01)^*^	−0.01 (0.01)	−0.01 (0.01)	−0.01 (0.01)
*R^2^*	0.13	0.17	0.05	0.16
*F* (df)	9.90^***^ (4, 260)	13.46^***^ (5, 259)	4.27^**^ (3, 261)	12.16^***^ (4, 260)
*F* vs. A(df)	(−-)	10.39^***^ (2, 258)	22.54^***^ (1, 259)	(−-)
*F* vs. B(df)	10.39^***^ (1, 258)	(−-)	32.93^**^ (2, 257)	3.67^*^ (1, 258)
AIC	725.48	715.17	748.33	717.59
BIC	746.95	740.23	766.24	739.06

Anxiety = (OXTR. rs2254298 = AA/AG) [B0 + B1(XMAST-C)] + (OXT. rs2254298 = GG) [B0 + B2 (XMAST-C)] + B3Xage + B4Xeducational years; F versus stands for F tests of the difference in *R*^2^ for a given model versus the robust differential susceptibility model; a parameter fixed at reported value; SE is not applicable, and is denoted as –.

## 4. Discussion

Based on the framework of G × E research on the etiology of alcohol-related mood disorders, we investigated the interaction effect between OXTR rs2254298 and alcohol dependence level on mood disorders in Han Chinese men during alcohol withdrawal. The alcohol dependence level had a primary effect on anxiety and depression. OXTR rs2254298 and alcohol dependence level significantly affected anxiety symptoms that were not present in depression. The interaction pattern between OXTR rs2254298 and alcohol dependence level fits the weak differential susceptibility model. The GG homozygote was a plasticity homozygote, and the A allele was a non-plasticity allele, suggesting that GG homozygote carriers are susceptible to the environment and likely to experience anxiety symptoms in adverse environments.

Consistent with previous studies (35, 36), we found that anxiety and depression symptoms during withdrawal in AUD patients are highly correlated with alcohol dependence levels in withdrawal. There is an established relationship between alcohol and anxiety; high anxiety levels in AUD patients manifest during alcohol withdrawal (37–39). Acute alcohol drinking stimulates gamma-aminobutyric acid (GABA) receptors, dampening brain activity and reducing anxiety (40). However, chronic alcohol consumption can lead to tolerance to GABA-ergic effects, and adaptation puts the brain into a constant state of anxiety and depression (40, 41); regional changes in nicotinic receptor function in the nucleus accumbens and ventral tegmental area have also been reported (42). Such decreases in reward system function may persist in adverse long-term biochemical changes contributing to the clinical syndrome of acute withdrawal and prolonged abstinence (43, 44). In alignment with a previous study, we showed that as alcohol dependence increases, the severity of anxiety and depression symptoms would also increase during alcohol withdrawal.

Hierarchical multiple regression revealed that the OXTR rs2254298 gene polymorphism was not directly associated with anxiety symptoms during withdrawal; however, the interaction between OXTR rs2254298 and alcohol dependence level had a significant effect on anxiety symptoms. The partial correlation value of 0.13 and half correlation value of 0.12 for the modulating effect of the OXTR SNP on anxiety that we reported can be considered small but meaningful effect sizes, with conversion calculations giving R2 of 0.017 and 0.014. According to Cohen (45), effect sizes R2 values of 0.01, 0.09 and 0.25 are considered small, medium and large, respectively. The R2 values in our study are 0.017 and 0.014, suggesting small but significant effects. Given that natural genetic variation typically exerts very subtle influences, these effect sizes are comparable to some previous single-locus investigations, suggesting similarly small but significant relationships. The RoS test and re-parameterized regression analysis indicated that the interaction of OXTR rs2254298 × current environment (alcohol dependence level) fits the weak differential susceptibility model. OXTR rs2554298 is associated with environmental sensitivity. GG homozygote is a plastic gene whose carriers are sensitive to environmental stress and more to anxiety symptoms in adverse environments.

OXT acts as an anxiolytic (44), and the OXTR is the cognate receptor for OXT (46). OXT is a stress hormone, and studies showed that stress induces an increase in oxytocin secretion (47–49). OXT is a neurotransmitter or neuromodulator with central actions in the limbic system, especially the amygdala, an essential structure in mood disorders (50). Slattery et al. reported modifications of neural activity induced by OXT in animal models of depression (51). Milrod et al. reported that altered plasma OXT levels are associated with more significant anxiety and relationship dissatisfaction in persons with separation anxiety disorder (52). Animal studies revealed that oxytocinergic circuits from the hypothalamus regulate GABA signaling, and decreased OXTR expression modulates presynaptic GABA release (53). These findings suggest that the OXTR modulates anxiety-related behaviors by affecting the excitability of branching GABA neurons. The OXTR participates in social processes and underlying traits of anxiety and depression, with little evidence for the effect of the OXTR SNPs in the etiology of clinical expressions of anxiety and depression. OXTR variation (rs53576) interacts with early threat exposure, with the threat-exposed rs53576 A allele demonstrating more significant emotion dysregulation (54). This finding suggests that OXTR SNP mutations are involved in emotion regulation.

We are also curious the reason why only association with anxiety rather than depression were found. Although anxiety and depression frequently co-occurring during withdrawal, these two kinds of mental disorders may be under distinct genetic control in some individuals. The oxytocin system specifically could have a stronger influence on withdrawal-related anxiety compared to depression. Oxytocin is strongly implicated in stress responses and modulation of the anxiety-mediating fight-or-flight sympathetic nervous system activation (55). As alcohol withdrawal represents a profound physiological stressor, OXTR genotypes that worsen oxytocin’s regulation of stress reactivity could intensify withdrawal-related anxiety, reflecting the immediate effects of the three-week acute withdrawal period. However, depression is a long-term accumulation of negative emotions that develops over time and may be more associated with other chronic and enduring factors. Therefore, individuals in the acute phase of alcohol withdrawal are more likely to experience heightened anxiety symptoms rather than depressive symptoms.

Because the OXTR rs2254298 is an intronic SNP, the mechanism by which the plastic homozygote works remains poorly understood. Several studies attempted to determine how the rs2554298 genotype regulates psychiatric symptoms. Regarding physiological mechanisms, Smearman et al. reported that rs2254298 GG carriers have more significant methylation at the cg11589699 site than AA/AG carriers (56), inhibiting the OXTR expression and reducing OXT levels (57). Lower levels of OXT are associated with more significant anxiety due to dysregulation of the hypothalamic–pituitary axis (58). Moreover, rs2254298 GG carriers exhibit lower attachment security than those with the A allele (59). According to attachment theory, individuals with insecure attachment exhibit more internalizing problems, such as emotional symptoms, and externalizing problems, such as behavioral problems and attention deficits in stressful situations (60). Thus, higher OXTR methylation and less secure attachment styles might be how OXTR rs2254298 regulates alcohol dependence and anxiety during alcohol withdrawal.

There are some limitations in this study. First, because of the size of G × E and the small sample size (single gender composition), these results should be interpreted cautiously, validated in larger samples, and compared with others. Second, our study examined only one OXTR candidate polymorphism in cross-section, which can be investigated by gene set analysis or pathway analysis in longitudinal studies (61). Third, the data including anxiety measures were self-report scales, and self-reporting bias was unavoidable (62). Furthermore, a specific tool designed explicitly for assessing withdrawal symptoms was not employed in the current study. However, the MAST contains several key items that could also aid in assessment of withdrawal symptoms especially among acute alcohol withdrawal patients who have already undergone mandatory detoxification for at least 3 weeks. Finally, though the current study inquired rudimentary information on patients’ smoking behavior (do you smoke; on average how many cigarettes do you smoke daily?). Specific assessment tools for smoking behavior should be employed to conduct in-depth to further investigate and distinguish possible effects of nicotine dependence and the role it plays in acute alcohol withdrawal and mood disorders.

Most previous studies focused on the interaction of individual early experiences with SNP (24). Our study investigated the interaction between OXTR rs2254298 and the current alcohol withdrawal environment on anxiety symptoms, providing evidence for the weak differential susceptibility model. Our findings help to elucidate the genetic basis for individual differences in negative emotions during alcohol withdrawal among alcohol-dependent patients. It is recommended that the proposed physiological and psychological mechanisms be validated in future studies.

## 5. Conclusion

The level of alcohol dependence correlates with anxiety risk in AUD patients, which may vary by OXTR genotypes. Specifically, rs2254298 GG carriers with AUD may have higher OXTR methylation, lower OXT levels, less secure attachment, and higher anxiety levels during alcohol withdrawal. These findings suggest that treatment for AUD patients with anxiety would be more effective when combined with pharmacological and psychological therapy (63), especially for the OXTR rs2254298 GG carriers with higher plasticity in the effect of the current environment.

## Data availability statement

The original contributions presented in the study are included in the article/Supplementary material, further inquiries can be directed to the corresponding authors.

## Ethics statement

The studies involving human participants were reviewed and approved by Peking University Health Science Center. The patients/participants provided their written informed consent to participate in this study. Written informed consent was obtained from the individual(s) for the publication of any potentially identifiable images or data included in this article.

## Author contributions

YL, FaW, and LC designed the study. GS, FaW, SY, and FeW contributed to data acquisition. GS, FaW, SY, and LW drafted the manuscript. GS, FeW, SY, LW, YC, YH, FZ, WW, PL, and LC participated in data analysis and interpretation. All authors read and approved the final manuscript.

## Funding

This study was supported by the Technology Support Project of Xinjiang (2017E0267, FaW), Natural Science Foundation of Xinjiang Uyghur Autonomous Region (2018D01C228, FaW), Tianshan Youth Project–Outstanding Youth Science and Technology Talents of Xinjiang (2017Q007, FaW), Beijing Natural Science Foundation (7152074, FaW), and the Opening Project of Zhejiang Provincial Top Key Discipline of Pharmaceutical Sciences (YL).

## Conflict of interest

The authors declare that the research was conducted in the absence of any commercial or financial relationships that could be construed as a potential conflict of interest.

## Publisher’s note

All claims expressed in this article are solely those of the authors and do not necessarily represent those of their affiliated organizations, or those of the publisher, the editors and the reviewers. Any product that may be evaluated in this article, or claim that may be made by its manufacturer, is not guaranteed or endorsed by the publisher.

## References

[1] Hu A Jiang H Dowling R Guo L Zhao X Hao W. The transition of alcohol control in China 1990-2019: impacts and recommendations. Int J Drug Policy. (2022) 105:103698. doi: 10.1016/j.drugpo.2022.103698, PMID: 35483250 PMC9247746

[2] Gonzalez E Azkargorta M Garcia-Vallicrosa C Prieto-Elordui J Elortza F Blanco-Sampascual S. Could protein content of urinary extracellular vesicles be useful to detect cirrhosis in alcoholic liver disease? Int J Biol Sci. (2021) 17:1864–77. doi: 10.7150/ijbs.59725, PMID: 34131392 PMC8193259

[3] Cheng HG Deng F Xiong W Phillips MR. Prevalence of alcohol use disorders in mainland China: a systematic review. Addiction. (2015) 110:761–74. doi: 10.1111/add.12876, PMID: 25678403 PMC6680273

[4] Hinton DJ Vázquez MS Geske JR Hitschfeld MJ Ho AMC Karpyak VM. Metabolomics biomarkers to predict Acamprosate treatment response in alcohol-dependent subjects. Sci Rep. (2017) 7:2496. doi: 10.1038/s41598-017-02442-4, PMID: 28566752 PMC5451388

[5] Joffe ME Centanni SW Jaramillo AA Winder DG Conn PJ. Metabotropic glutamate receptors in alcohol use disorder: physiology, plasticity, and promising pharmacotherapies. ACS Chem Neurosci. (2018) 9:2188–204. doi: 10.1021/acschemneuro.8b00200, PMID: 29792024 PMC6192262

[6] Oliva F Nibbio G Vizzuso P Jaretti Sodano A Ostacoli L Carletto S. Gender differences in anxiety and depression before and after alcohol detoxification: anxiety and depression as gender-related predictors of relapse. Eur Addict Res. (2018) 24:163–72. doi: 10.1159/000490046, PMID: 30016803 PMC6172794

[7] Hershon HI. Alcohol withdrawal symptoms: phenomenology and implications. Br J Addict Alcohol Other Drugs. (1973) 68:295–302. doi: 10.1111/j.1360-0443.1973.tb01260.x, PMID: 4606187

[8] Rasmussen DD Boldt BM Bryant CA Mitton DR Larsen SA Wilkinson CW. Chronic daily ethanol and withdrawal: 1. Long-term changes in the Hypothalamo-pituitary-adrenal Axis. Alcohol Clin Exp Res. (2000) 24:1836–49. doi: 10.1111/j.1530-0277.2000.tb01988.x, PMID: 11141043

[9] Heinrichs SC Koob GF. Corticotropin-releasing factor in brain: a role in activation, arousal, and affect regulation. J Pharmacol Exp Ther. (2004) 311:427–40. doi: 10.1124/jpet.103.052092, PMID: 15297468

[10] Fu R Mei Q Shiwalkar N Zuo W Zhang H Gregor D. Anxiety during alcohol withdrawal involves 5-Ht2c receptors and M-channels in the lateral Habenula. Neuropharmacology. (2020) 163:107863. doi: 10.1016/j.neuropharm.2019.107863, PMID: 31778691

[11] Agostini JF Costa NLF Bernardo HT Baldin SL Mendes NV de Pieri PK. Ceftriaxone attenuated anxiety-like behavior and enhanced brain glutamate transport in zebrafish subjected to alcohol withdrawal. Neurochem Res. (2020) 45:1526–35. doi: 10.1007/s11064-020-03008-z, PMID: 32185643

[12] Walker LC Kastman HE Krstew EV Gundlach AL Lawrence AJ. Central amygdala Relaxin-3/Relaxin family peptide receptor 3 Signalling modulates alcohol seeking in rats. Br J Pharmacol. (2017) 174:3359–69. doi: 10.1111/bph.13955, PMID: 28726252 PMC5595761

[13] Schuckit MA Danko GP Smith TL Hesselbrock V Kramer J Bucholz K. A 5-year prospective evaluation of Dsm-iv alcohol dependence with and without a physiological component. Alcohol Clin Exp Res. (2003) 27:818–25. doi: 10.1097/01.Alc.0000067980.18461.33, PMID: 12766627

[14] Rutter M. Gene-Environment Interdependence. Dev Sci. (2007) 10:12–8. doi: 10.1111/j.1467-7687.2007.00557.x 17181693

[15] Belsky J Pluess M Widaman KF. Confirmatory and competitive evaluation of alternative gene-environment interaction hypotheses. J Child Psychol Psychiatry. (2013) 54:1135–43. doi: 10.1111/jcpp.12075, PMID: 23617948

[16] Bach P Koopmann A Bumb JM Zimmermann S Bühler S Reinhard I. Oxytocin attenuates neural response to emotional faces in social drinkers: an Fmri study. Eur Arch Psychiatry Clin Neurosci. (2021) 271:873–82. doi: 10.1007/s00406-020-01115-0, PMID: 32076819 PMC8236029

[17] Lee HJ Macbeth AH Pagani JH Young WS 3rd. Oxytocin: the great facilitator of life. Prog Neurobiol. (2009) 88:127–51. doi: 10.1016/j.pneurobio.2009.04.001, PMID: 19482229 PMC2689929

[18] Kosfeld M Heinrichs M Zak PJ Fischbacher U Fehr E. Oxytocin increases Trust in Humans. Nature. (2005) 435:673–6. doi: 10.1038/nature03701 15931222

[19] Lang RE Heil JW Ganten D Hermann K Unger T Rascher W. Oxytocin unlike vasopressin is a stress hormone in the rat. Neuroendocrinology. (1983) 37:314–6. doi: 10.1159/000123566, PMID: 6633820

[20] Israel S Lerer E Shalev I Uzefovsky F Reibold M Bachner-Melman R. Molecular genetic studies of the arginine vasopressin 1a receptor (Avpr1a) and the oxytocin receptor (Oxtr) in human behaviour: from autism to altruism with some notes in between. Prog Brain Res. (2008) 170:435–49. doi: 10.1016/s0079-6123(08)00434-2, PMID: 18655900

[21] Toepfer P Heim C Entringer S Binder E Wadhwa P Buss C. Oxytocin pathways in the intergenerational transmission of maternal early life stress. Neurosci Biobehav Rev. (2017) 73:293–308. doi: 10.1016/j.neubiorev.2016.12.026, PMID: 28027955 PMC5272812

[22] Flasbeck V Moser D Kumsta R Brüne M. The Oxtr single-nucleotide polymorphism Rs53576 moderates the impact of childhood maltreatment on empathy for social pain in female participants: evidence for differential susceptibility. Front Psych. (2018) 9:359. doi: 10.3389/fpsyt.2018.00359, PMID: 30135663 PMC6092568

[23] Uzefovsky F Bethlehem RAI Shamay-Tsoory S Ruigrok A Holt R Spencer M. The oxytocin receptor gene predicts brain activity during an emotion recognition task in autism. Mol Autism. (2019) 10:12. doi: 10.1186/s13229-019-0258-4, PMID: 30918622 PMC6419364

[24] Thompson RJ Parker KJ Hallmayer JF Waugh CE Gotlib IH. Oxytocin receptor gene polymorphism (Rs2254298) interacts with familial risk for psychopathology to predict symptoms of depression and anxiety in adolescent girls. Psychoneuroendocrinology. (2011) 36:144–7. doi: 10.1016/j.psyneuen.2010.07.003, PMID: 20708845 PMC2997902

[25] Ebstein RP Israel S Chew SH Zhong S Knafo A. Genetics of human social behavior. Neuron. (2010) 65:831–44. doi: 10.1016/j.neuron.2010.02.020 20346758

[26] Chelala C Khan A Lemoine NR. Snpnexus: a web database for functional annotation of newly discovered and public domain single nucleotide polymorphisms. Bioinformatics. (2009) 25:655–61. doi: 10.1093/bioinformatics/btn653, PMID: 19098027 PMC2647830

[27] Wade M Hoffmann TJ Wigg K Jenkins JM. Association between the oxytocin receptor (Oxtr) gene and Children's social cognition at 18 months. Genes Brain Behav. (2014) 13:603–10. doi: 10.1111/gbb.12148, PMID: 24916666

[28] Camerini L Zurchimitten G Bock B Xavier J Bastos CR Martins E. Genetic variations in elements of the Oxytocinergic pathway are associated with attention/hyperactivity problems and anxiety problems in childhood. Child Psychiatry Hum Dev. (2022) 2022:1419. doi: 10.1007/s10578-022-01419-3, PMID: 36087156

[29] Storgaard H Nielsen SD Gluud C. The validity of the Michigan alcoholism screening test (Mast). Alcohol Alcohol. (1994) 29:493–502. PMID: 7811333

[30] Skinner HA Allen BA. Alcohol dependence syndrome: measurement and validation. J Abnorm Psychol. (1982) 91:199–209. doi: 10.1037//0021-843x.91.3.199 7096790

[31] Dunstan DA Scott N. Norms for Zung's self-rating anxiety scale. BMC Psychiatry. (2020) 20:90. doi: 10.1186/s12888-019-2427-6 32111187 PMC7048044

[32] Soósová MS Macejová Ž Zamboriová M Dimunová L. Anxiety and depression in Slovak patients with rheumatoid arthritis. J Ment Health (Abingdon, England). (2017) 26:21–7. doi: 10.1080/09638237.2016.1244719, PMID: 27809630

[33] Brüne M. Does the oxytocin receptor (Oxtr) polymorphism (Rs2254298) confer 'Vulnerability' for psychopathology or 'Differential Susceptibility'? Insights from evolution. BMC Med. (2012) 10:38. doi: 10.1186/1741-7015-10-38, PMID: 22510359 PMC3386011

[34] Zhang J Yang C Leng J Liu J Gong P Esposito G. Oxtr moderates adverse childhood experiences on depressive symptoms among incarcerated males. J Psychiatr Res. (2021) 140:221–7. doi: 10.1016/j.jpsychires.2021.05.043, PMID: 34118640

[35] Wei X Cai F Zhou S Zhang J Xu K Shen G. The neuropeptide Y single-nucleotide polymorphism Rs16147:T>C moderates the effect of alcohol dependence on depression in male Chinese Han population. Front Psych. (2022) 13:1012850. doi: 10.3389/fpsyt.2022.1012850, PMID: 36245887 PMC9558829

[36] Hong L Wen L Niculescu M Zhou F Zou Y Shen G. The interaction between Pomc Rs2071345 polymorphism and alcohol dependence in anxiety symptoms among Chinese male problem drinkers. Front Psych. (2022) 13:878960. doi: 10.3389/fpsyt.2022.878960, PMID: 35592377 PMC9110641

[37] Blanchard RJ Magee L Veniegas R Blanchard DC. Alcohol and anxiety: Ethopharmacological approaches. Prog Neuro-Psychopharmacol Biol Psychiatry. (1993) 17:171–82. doi: 10.1016/0278-5846(93)90041-p, PMID: 8094255

[38] Gilpin NW Herman MA Roberto M. The central amygdala as an integrative hub for anxiety and alcohol use disorders. Biol Psychiatry. (2015) 77:859–69. doi: 10.1016/j.biopsych.2014.09.008, PMID: 25433901 PMC4398579

[39] Anker JJ Kushner MG. Co-occurring alcohol use disorder and anxiety: bridging psychiatric, psychological, and neurobiological perspectives. Alcohol Res. (2019) 40:3. doi: 10.35946/arcr.v40.1.03, PMID: 31886106 PMC6927748

[40] Koob GF Volkow ND. Neurocircuitry of addiction. Neuropsychopharmacology. (2010) 35:217–38. doi: 10.1038/npp.2009.110, PMID: 19710631 PMC2805560

[41] Koob GF Volkow ND. Neurobiology of addiction: a Neurocircuitry Analysis. Lancet Psychiatry. (2016) 3:760–73. doi: 10.1016/s2215-0366(16)00104-8, PMID: 27475769 PMC6135092

[42] Tolu S Eddine R Marti F David V Graupner M Pons S. Co-activation of Vta Da and Gaba neurons mediates nicotine reinforcement. Mol Psychiatry. (2013) 18:382–93. doi: 10.1038/mp.2012.83, PMID: 22751493

[43] Delfs JM Zhu Y Druhan JP Aston-Jones G. Noradrenaline in the ventral forebrain is critical for opiate withdrawal-induced aversion. Nature. (2000) 403:430–4. doi: 10.1038/35000212, PMID: 10667795

[44] Carlezon WA Nestler EJ Neve RL. Herpes simplex virus-mediated gene transfer as a tool for neuropsychiatric research. Crit Rev Neurobiol. (2000) 14:47–67. doi: 10.1080/08913810008443546, PMID: 11253955

[45] Statistical CJ Analysis P. Curr Dir Psychol Sci. (1992) 1:98–101. doi: 10.1111/1467-8721.ep10768783

[46] Neumann ID Slattery DA. Oxytocin in general anxiety and social fear: a translational approach. Biol Psychiatry. (2016) 79:213–21. doi: 10.1016/j.biopsych.2015.06.004 26208744

[47] Takayanagi Y Onaka T. Roles of oxytocin in stress responses, Allostasis and resilience. Int J Mol Sci. (2021) 23:150. doi: 10.3390/ijms23010150, PMID: 35008574 PMC8745417

[48] Carter CS Kenkel WM MacLean EL Wilson SR Perkeybile AM Yee JR. Is oxytocin “nature’s medicine”? Pharmacol Rev. (2020) 72:829–61. doi: 10.1124/pr.120.019398, PMID: 32912963 PMC7495339

[49] Young Kuchenbecker S Pressman SD Celniker J Grewen KM Sumida KD Jonathan N. Oxytocin, cortisol, and cognitive control during acute and naturalistic stress. Stress. (2021) 24:370–83. doi: 10.1080/10253890.2021.1876658, PMID: 33632072 PMC8254750

[50] Costa B Pini S Baldwin DS Silove D Manicavasagar V Abelli M. Oxytocin receptor and G-protein polymorphisms in patients with depression and separation anxiety. J Affect Disord. (2017) 218:365–73. doi: 10.1016/j.jad.2017.03.056 28499211

[51] Slattery DA Neumann ID. Oxytocin and major depressive disorder: experimental and clinical evidence for links to aetiology and possible treatment. Pharmaceuticals (Basel). (2010) 3:702–24. doi: 10.3390/ph3030702, PMID: 27713275 PMC4033976

[52] Milrod B Markowitz JC Gerber AJ Cyranowski J Altemus M Shapiro T. Childhood separation anxiety and the pathogenesis and treatment of adult anxiety. Am J Psychiatry. (2014) 171:34–43. doi: 10.1176/appi.ajp.2013.13060781 24129927

[53] Krueger F Parasuraman R Iyengar V Thornburg M Weel J Lin M. Oxytocin receptor genetic variation promotes human trust behavior. Front Hum Neurosci. (2012) 6:4. doi: 10.3389/fnhum.2012.00004, PMID: 22347177 PMC3270329

[54] Ripamonti S Ambrozkiewicz MC Guzzi F Gravati M Biella G Bormuth I. Transient oxytocin signaling primes the development and function of excitatory hippocampal neurons. Elife. (2017) 6:e22466. doi: 10.7554/eLife.22466, PMID: 28231043 PMC5323041

[55] Campbell A Hausmann M. Effects of oxytocin on Women's aggression depend on state anxiety. Aggress Behav. (2013) 39:316–22. doi: 10.1002/ab.21478, PMID: 23553462

[56] Smearman EL Almli LM Conneely KN Brody GH Sales JM Bradley B. Oxytocin receptor genetic and epigenetic variations: association with child abuse and adult psychiatric symptoms. Child Dev. (2016) 87:122–34. doi: 10.1111/cdev.12493, PMID: 26822448 PMC4733885

[57] Klose RJ Bird AP. Genomic DNA methylation: the mark and its mediators. Trends Biochem Sci. (2006) 31:89–97. doi: 10.1016/j.tibs.2005.12.008 16403636

[58] Uchino BN Way BM. Integrative pathways linking close family ties to health: a neurochemical perspective. Am Psychol. (2017) 72:590–600. doi: 10.1037/amp0000049, PMID: 28880105

[59] Chen FS Barth ME Johnson SL Gotlib IH Johnson SC. Oxytocin receptor (Oxtr) polymorphisms and attachment in human infants. Front Psychol. (2011) 2:200. doi: 10.3389/fpsyg.2011.00200, PMID: 21904531 PMC3161247

[60] Cook SH Valera P Calebs BJ Wilson PA. Adult attachment as a moderator of the association between childhood traumatic experiences and depression symptoms among Young black gay and bisexual men. Cultur Divers Ethnic Minor Psychol. (2017) 23:388–97. doi: 10.1037/cdp0000119, PMID: 27736103 PMC5391313

[61] Dunn EC Soare TW Zhu Y Simpkin AJ Suderman MJ Klengel T. Sensitive periods for the effect of childhood adversity on DNA methylation: results from a prospective, longitudinal study. Biol Psychiatry. (2019) 85:838–49. doi: 10.1016/j.biopsych.2018.12.023 30905381 PMC6552666

[62] Motizuki M Kohno H Tsurugi K. A study on the identities of the three species of chromatin-associated proteinases in a mutant of *Saccharomyces cerevisiae* which lacks four major vacuolar proteinases. J Biochem. (1988) 104:192–5. doi: 10.1093/oxfordjournals.jbchem.a122440 3053680

[63] Schuckit MA. Alcohol-use disorders. Lancet. (2009) 373:492–501. doi: 10.1016/s0140-6736(09)60009-x 19168210

